# Diagnostic accuracy of neuropathological assessment across different diagnostic settings for high-grade gliomas: the added value of DNA methylation profiling

**DOI:** 10.1007/s00701-026-07078-w

**Published:** 2026-10-24

**Authors:** Timotheos Christoforou, Umida Elmuradova, Stefanie Hammersen, Arend Koch, Sven Kantelhardt, Ali Mulhem

**Affiliations:** 1 Department of Neurosurgery, Vivantes Klinikum Im Friedrichshain, Berlin, Germany; 2 Department for Continuing Education, University of Oxford, Oxford, UK; 3 Department of Neurosurgery, Helios Klinikum Erfurt, Erfurt, Germany; 4 Department of Neuropathology, Charité – Universitätsmedizin Berlin, Berlin, Germany

**Keywords:** High-grade glioma, DNA methylation profiling, Neuropathology, Diagnostic accuracy, Brain tumor

## Abstract

**Background:**

Accurate histopathological diagnosis is essential for the management of patients undergoing surgery for suspected high-grade glioma (HGG). We evaluated the diagnostic accuracy of pathological assessment performed in a general pathology department and a specialist neuropathology department, using EPIC-based DNA methylation classification as the operational reference standard.

**Methods:**

This retrospective diagnostic accuracy study included 130 patients with complete results from three diagnostic assessments (selected from 173 screened records) with intracranial space-occupying lesions who underwent surgical biopsy or resection between May 2019 and February 2025. Tissue specimens were assessed in a general pathology department and subsequently evaluated independently in a specialist neuropathology department, followed by DNA methylation profiling using the Illumina Infinium MethylationEPIC BeadChip. HGGs (CNS WHO grades 3–4) were classified as positive and all other lesions as negative.

**Results:**

Assessment in the general pathology department yielded 20 HGG classifications discordant with the EPIC reference (false negatives), including 13 tumors initially classified as low-grade gliomas, two as other intracranial lesions, and five as non-neoplastic brain tissue. Sensitivity was 79%, specificity 100%, positive predictive value 100%, negative predictive value 62%, and overall accuracy 84%. Specialist neuropathological assessment achieved a sensitivity of 100%, specificity of 97%, positive predictive value of 99%, negative predictive value of 100%, and overall accuracy of 99%. Three cases differed in specific classification or binary status: two HGG diagnoses were further refined as glioblastoma, IDH-wildtype, CNS WHO grade 4, while one specimen interpreted histopathologically as showing proliferative and mitotic activity was classified as tumor-free brain tissue by DNA methylation profiling.

**Conclusions:**

Specialist neuropathological assessment showed high agreement with the EPIC-based reference classification for HGGs, whereas assessment outside a specialized neuropathology setting showed lower sensitivity for HGG identification in this selected cohort. DNA methylation profiling provides complementary diagnostic value by refining tumour classification.

## Introduction

Gliomas are the most common primary tumours of the central nervous system (CNS) and are thought to arise from neural stem or progenitor cells that acquire tumour-initiating genetic alterations [29]. They are classified according to their histopathological and molecular characteristics, as outlined in the World Health Organization (WHO) Classification of Tumours of the Central Nervous System [19]. Gliomas are assigned CNS WHO grades 1–4, reflecting increasing degrees of biological aggressiveness and malignancy.

Over the past decade, numerous molecular and genetic diagnostic techniques have been introduced to complement conventional histopathological assessment, including microscopic examination and immunohistochemical analysis of intracranial tumour specimens [7, 26]. Among these, DNA methylation profiling has emerged as an important molecular diagnostic approach for the classification of CNS tumours [3].

The current WHO Classification of CNS Tumours requires assessment of several molecular alterations for the classification of many intracranial tumours, including gliomas [19]. Nevertheless, histopathological assessment remains the foundation of brain tumour diagnosis and the primary diagnostic approach in many centres. Its diagnostic performance, however, may depend on the expertise of the evaluating pathology service, particularly for CNS tumours with overlapping or subtle morphological features. This distinction is clinically relevant because tissue obtained during neurosurgical biopsy or resection may initially be evaluated either within a general pathology service or at a centre with dedicated neuropathological expertise.

Advanced molecular techniques such as DNA methylation profiling can improve diagnostic precision and facilitate the classification of diagnostically challenging CNS tumours but are associated with additional costs, limited availability, and potentially longer turnaround times [13, 27, 33]. For patients with high-grade gliomas, timely establishment of the diagnosis is particularly important because delays may postpone the initiation of appropriate oncological treatment [2, 6, 12, 16, 21, 24, 34].

DNA methylation profiling may also lead to refinement or reclassification of tumours initially diagnosed using conventional histopathological methods [3, 13, 15, 32]. Such diagnostic discordance raises two clinically relevant questions: how reliably conventional pathological assessment identifies high-grade gliomas and whether diagnostic performance differs between general pathology and specialist neuropathology settings. Furthermore, even when specialist neuropathology correctly identifies a tumour as high grade, DNA methylation profiling may provide additional information relevant to the final integrated diagnosis.

Comparative evidence addressing these different components of the diagnostic pathway remains limited. A better understanding of the performance of conventional pathological assessment across different institutional diagnostic settings, and of the additional diagnostic contribution of DNA methylation profiling, may help optimize the evaluation of tissue obtained during neurosurgical procedures. Therefore, this study aimed to compare the diagnostic accuracy of conventional pathological assessment performed in a general pathology department and a specialist neuropathology department for identifying high-grade gliomas, using DNA methylation profiling as the operational reference standard. We additionally evaluated cases in which DNA methylation profiling refined or differed from the conventional pathological diagnosis.

## Methods

### Study design

We conducted a retrospective diagnostic accuracy study of patients diagnosed with intracranial space-occupying lesions (ICSOLs) between May 2019 and February 2025. The initial diagnosis of an ICSOL was established using magnetic resonance imaging (MRI). Patients underwent surgical resection or biopsy, followed by neuropathological evaluation. Eligible patients were identified retrospectively based on the availability of complete archived results from two independent neuropathological assessments and DNA methylation profiling. This study is reported in accordance with the Standards for Reporting Diagnostic Accuracy Studies (STARD 2015) guideline.

Three diagnostic assessments were compared. Index Test I consisted of conventional neuropathological evaluation, including histopathological examination and immunohistochemistry, performed by an experienced neuropathologist at the Department of Pathology, Vivantes Klinikum im Friedrichshain, Berlin, Germany, where the reporting neuropathologist served as head of the general pathology department. This department had microscopic and immunohistochemical facilities but no access to molecular or genetic diagnostic tools. Index Test II consisted of an independent neuropathological evaluation, including histopathology and immunohistochemistry, performed at the Department of Neuropathology, Charité–Universitätsmedizin Berlin, Germany. Both index assessments were performed by neuropathologists; the comparison therefore concerns different institutional diagnostic settings rather than different professional qualifications. The operational reference standard for the diagnostic accuracy analysis was EPIC-based DNA methylation profiling performed using the Illumina Infinium MethylationEPIC BeadChip (EPIC array) at the Department of Neuropathology, Charité–Universitätsmedizin Berlin, as part of the routine diagnostic workflow. EPIC-based classifications, rather than independently adjudicated integrated diagnoses, formed the reference categories used in this analysis. DNA methylation profiling was not selectively performed for this study on the basis of diagnostic uncertainty or discordance between the pathological assessments.

The two institutions performed their neuropathological evaluations independently and were blinded to each other's findings. Index Test I and Index Test II were performed on distinct tissue samples from the same tumour. DNA methylation profiling was performed at the same institution as Index Test II and used the same tissue specimen as that histopathological assessment. However, the Index Test II report was finalised and the diagnosis recorded before the results of DNA methylation profiling became available. Thus, the neuropathologists reporting Index Test II were blinded to the methylation profiling results at the time of their assessment. Although this temporal blinding reduced the risk of diagnostic review bias, the use of the same tissue specimen for Index Test II and DNA methylation profiling, in contrast to the separate specimen used for Index Test I, may have favoured agreement between Index Test II and the reference standard.

### Inclusion and exclusion criteria

We screened all patient records coded as intracranial space-occupying lesions between May 2019 and February 2025. Patients were included if they (1) underwent surgical resection or biopsy for an MRI-diagnosed ICSOL; (2) had complete archived results from conventional neuropathological assessment at our institution (Index Test I); and (3) had complete archived results from independent specialist neuropathological assessment (Index Test II) and DNA methylation profiling at Charité–Universitätsmedizin Berlin.

### Statistical analysis

For the primary diagnostic accuracy analysis, tumours were dichotomised into high-grade gliomas (CNS WHO grades 3 and 4) and non-high-grade lesions, the latter comprising low-grade gliomas (CNS WHO grades 1 and 2) and all other intracranial space-occupying lesions. High-grade gliomas were classified as positive cases, whereas all remaining lesions were classified as negative cases.

The diagnostic performance of Index Test I and Index Test II was evaluated separately using DNA methylation profiling as the reference standard. For each index test, discrepancies with the reference standard were summarised descriptively, and the numbers of true-positive, true-negative, false-positive, and false-negative results were determined. Based on these classifications, sensitivity, specificity, positive predictive value (PPV), negative predictive value (NPV), overall diagnostic accuracy, and F1 score were calculated together with their corresponding 95% confidence intervals (95% CIs), which were estimated using the Wilson score method.

All statistical analyses were performed using R statistical software (version 4.4.1; R Foundation for Statistical Computing, Vienna, Austria).

## Results

### Patient characteristics

**Fig. 1:**
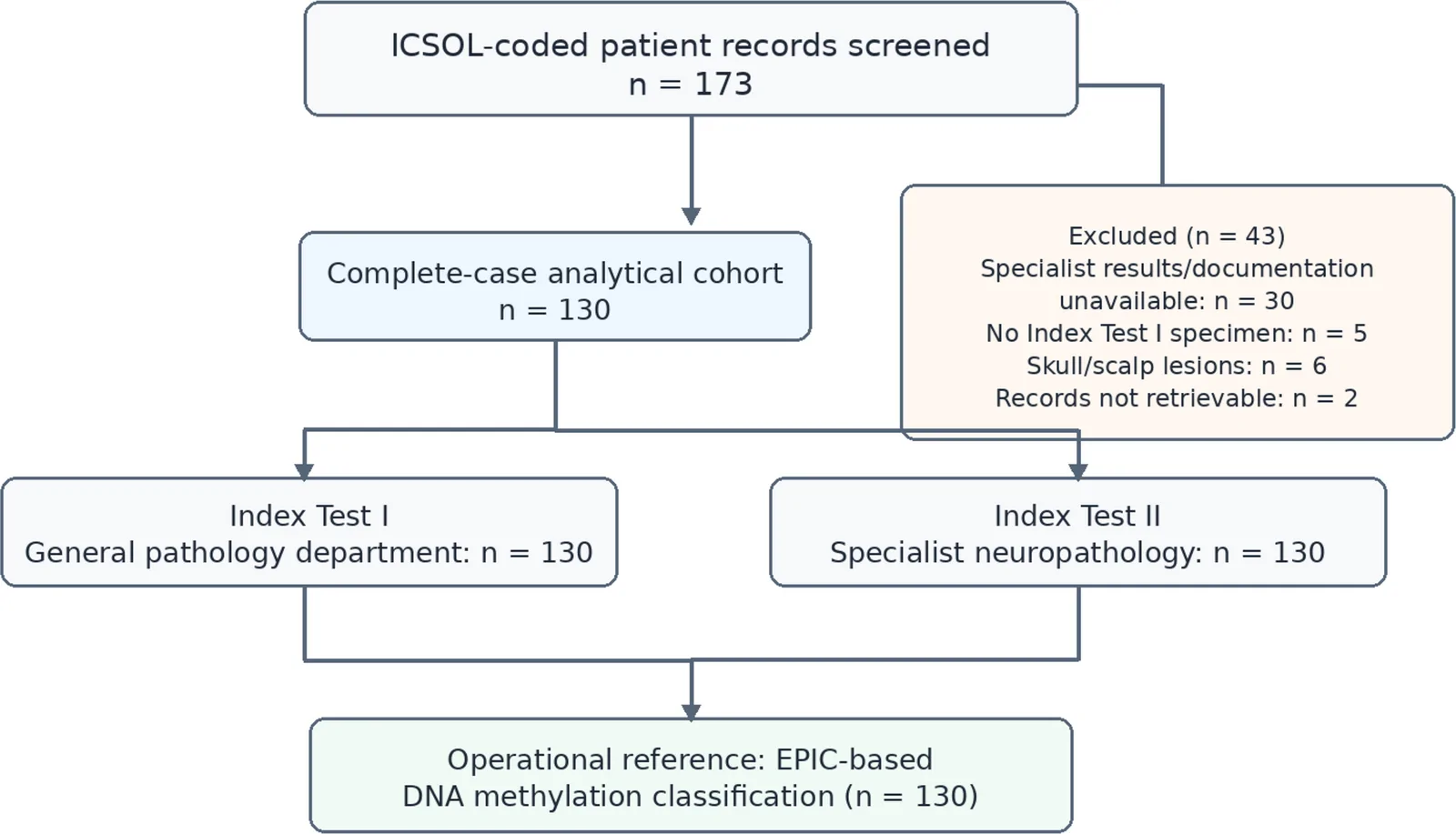
Patient selection flow diagram

Of the 173 screened records, 43 were excluded: 30 because complete diagnostic documentation from the specialist neuropathology department (Index Test II and/or DNA methylation profiling) was unavailable, five because no specimen had been submitted for Index Test I, six because subsequent review revealed skull or scalp tumours incorrectly coded as ICSOLs, and two because the relevant records could not be retrieved. The final analytical cohort comprised 130 patients with complete results from all three diagnostic assessments. The patient-selection process is illustrated in the study flowchart (Fig. 1). The mean patient age was 58 years (range, 11–86 years), and 49 patients (37.7%) were female.

### Diagnostic performance of index test I

Compared with DNA methylation profiling (reference standard), conventional neuropathological evaluation at Institution 1 (Index Test I) yielded 20 false-negative cases and no false positives.

Among the false-negative cases, 13 were initially diagnosed as low-grade gliomas, 2 as other intracranial space-occupying lesions, and 5 as non-neoplastic brain tissue showing nonspecific pathological changes without definitive evidence of tumour. The clinical and pathological characteristics of these discordant cases are presented in Table 1.

**Table 1:** Characteristics of the 20 false-negative cases identified by conventional neuropathological evaluation at Institution 1 (Index Test I) compared with DNA methylation profiling (reference standard)

Case	Age (years)	Sex	Tumour location	Index Test I diagnosis	Reference diagnosis
Initially interpreted as non-neoplastic brain tissue					
1	52	M	Left thalamus	Brain tissue with increased cell density	Diffuse midline glioma, H3 K27-altered (CNS WHO grade 4)
2	70	F	Left parietal	Tumour-free brain tissue	Glioblastoma, IDH-wildtype (CNS WHO grade 4)
3	63	M	Left posterior horn	Tumour-free brain tissue	Glioblastoma, IDH-wildtype (CNS WHO grade 4)
4	59	M	Left temporal	Brain tissue with chronic inflammation and gliosis	Glioblastoma, IDH-wildtype (CNS WHO grade 4)
5	54	F	Left precentral	Brain tissue fragments with radiation necrosis	Glioblastoma, IDH-wildtype (CNS WHO grade 4)
Initially diagnosed as low-grade glioma					
6	56	M	Left parieto-occipital	Astrocytoma (CNS WHO grade 2)	Glioblastoma, IDH-wildtype (CNS WHO grade 4)
7	45	M	Left occipital	Low-grade glioma	Oligodendroglioma, IDH-mutant, 1p/19q-codeleted (CNS WHO grade 3)
8	57	M	Left frontal	Low-grade glioma	Glioblastoma, IDH-wildtype (CNS WHO grade 4)
9	58	M	Left postcentral	Low-grade glioma	Glioblastoma, IDH-wildtype (CNS WHO grade 4)
10	74	F	Right cingulate gyrus	Low-grade glioma	Glioblastoma, IDH-wildtype (CNS WHO grade 4)
11	19	M	Left frontal	Low-grade glioma (CNS WHO grade 2)	Glioblastoma, IDH-wildtype (CNS WHO grade 4)
12	32	M	Right frontal	Low-grade glioma, most consistent with diffuse astrocytoma (CNS WHO grade 2)	Astrocytoma, IDH-mutant (CNS WHO grade 4)
13	60	F	Left temporal	Diffuse astrocytoma (CNS WHO grade 2)	Glioblastoma, IDH-wildtype (CNS WHO grade 4)
14	55	F	Medulla oblongata	Diffuse astrocytoma (at least CNS WHO grade 2)	Diffuse midline glioma, H3 K27M-altered (CNS WHO grade 4)
15	65	F	Right temporal	Diffuse astrocytoma (CNS WHO grade 2)	Glioblastoma, IDH-wildtype (CNS WHO grade 4)
16	38	M	Right temporal	Diffuse astrocytoma (CNS WHO grade 2)	Diffuse astrocytoma, IDH-wildtype with molecular features of glioblastoma (CNS WHO grade 4)
17	64	M	Right frontal	Low-grade glioma	Oligodendroglioma, IDH-mutant, 1p/19q-codeleted (CNS WHO grade 3)
18	75	F	Left frontoparietal	Low-grade glioma	Glioblastoma, IDH-wildtype (CNS WHO grade 4)
Initially diagnosed as another intracranial lesion					
19	58	M	Left precentral	High-grade lymphoma	Glioblastoma, IDH-wildtype (CNS WHO grade 4)
20	64	M	Left thalamus	Poorly differentiated malignant cellular neoplasm (consistent with metastatic melanoma)	High-grade astrocytoma with piloid features (assigned CNS WHO grade 3)

*CNS* central nervous system, *IDH* isocitrate dehydrogenase

Conventional neuropathology demonstrated a sensitivity of 79% (95% CI, 71%–87%) and a specificity of 100% for identifying high-grade gliomas, with no false-positive diagnoses. The positive predictive value (PPV) was 100%, whereas the negative predictive value (NPV) was 62% (95% CI, 48%–75%). The overall diagnostic accuracy was 84% (95% CI, 78%–91%), and the F1 score was 88% (95% CI, 82%–95%).

### Diagnostic performance of index test II

Overall, a high degree of concordance was observed between conventional neuropathological evaluation at Institution 2 (Index Test II) and DNA methylation profiling.

Three discrepant cases were identified (Table 2). One case, initially interpreted as CNS tissue demonstrating limited proliferative activity with a mitotic figure, was classified by DNA methylation profiling as tumour-free brain tissue, representing the only false-positive classification relative to the EPIC-based reference for Index Test II.

**Table 2:** Characteristics of the discrepant cases between conventional neuropathological evaluation at Institution 2 (Index Test II) and DNA methylation profiling (reference standard)

Case	Age (years)	Sex	Tumour location	Index Test II diagnosis	Reference diagnosis
Histopathological diagnosis refined by DNA methylation profiling					
1	65	F	Right temporal	Anaplastic astrocytoma	Glioblastoma, IDH-wildtype (CNS WHO grade 4)
2	56	M	Left parieto-occipital	Diffuse astroglial tumour with increased mitotic and proliferative activity	Glioblastoma, IDH-wildtype (CNS WHO grade 4)
False-positive conventional neuropathological diagnosis					
3	58	F	Right temporal	CNS tissue demonstrating limited proliferative activity with the presence of a mitotic figure	Tumour-free brain tissue

*CNS* central nervous system, *IDH* isocitrate dehydrogenase

In addition, two tumours initially classified by conventional neuropathology as high-grade astrocytic neoplasms were classified by DNA methylation profiling as glioblastoma, IDH-wildtype (CNS WHO grade 4). Specifically, one case diagnosed as an anaplastic astrocytoma and another diagnosed as a diffuse astroglial tumour with increased mitotic and proliferative activity were reclassified following molecular analysis. Both cases remained classified as HGGs in the binary diagnostic accuracy analysis; the study did not assess whether these refinements affected clinical management.

Using DNA methylation profiling as the operational reference standard, Index Test II demonstrated a sensitivity of 100%, a specificity of 97%, a positive predictive value (PPV) of 99%, a negative predictive value (NPV) of 100%, an overall diagnostic accuracy of 99%, and an F1 score of 99.5%.

## Discussion

This diagnostic accuracy study is designed to compare conventional pathological assessment performed in a general pathology department and a specialist neuropathology department with DNA methylation profiling for the identification of high-grade gliomas. Two principal findings emerged. First, pathological assessment performed in a general pathology setting showed only moderate sensitivity for identifying high-grade gliomas, despite excellent specificity. Notably, the assessment was performed by an experienced neuropathologist, but without access to the broader molecular diagnostic infrastructure available at a specialist neuropathology centre. Second, conventional assessment performed in a specialist neuropathology department demonstrated very high agreement with the EPIC-based reference classification. Nevertheless, DNA methylation profiling further refined the tumour classification in selected cases. Although institutional setting and tissue sampling cannot be disentangled in this study, these findings support specialist review of diagnostically challenging intracranial tumour specimens and indicate that DNA methylation profiling provides complementary diagnostic value even when conventional specialist neuropathological assessment is highly reliable.

Index Test I, performed in a general pathology department without access to further neuropathological molecular diagnostics, demonstrated a sensitivity of 79% and a specificity of 100% for the identification of high-grade gliomas. Although all cases classified as high-grade gliomas were confirmed by DNA methylation profiling, 20 cases classified as high-grade gliomas by EPIC were classified as non-high-grade lesions by conventional assessment. The positive predictive value was 100%, indicating that a positive high-grade glioma diagnosis was highly reliable. By contrast, the negative predictive value was only 62%, meaning that approximately 38% of Index Test I-negative lesions were classified as high-grade gliomas by the EPIC-based reference. Overall accuracy was 84%, with an F1 score of 88%.

The false-negative cases identified by Index Test I are clinically important. Five tumours were initially interpreted as non-neoplastic brain tissue, including specimens described as tumour-free, gliotic or chronically inflamed tissue, tissue with increased cellularity, or radiation necrosis. Thirteen additional tumours were initially classified as low-grade gliomas, and two were interpreted as other intracranial lesions. These findings illustrate the diagnostic challenges associated with limited tissue sampling, subtle histological features, treatment-related changes, and morphological overlap among intracranial lesions. Importantly, the findings should not be interpreted as reflecting pathological expertise alone, because Index Test I was assessed by an experienced neuropathologist. Rather, they highlight the potential value of a specialised diagnostic environment in which dedicated neuropathological assessment can be integrated with additional molecular investigations when required. From a neurosurgical perspective, this is particularly relevant when histopathological findings appear discordant with the radiological or intraoperative suspicion of an aggressive tumour.

In contrast, Index Test II, performed in a specialist neuropathology department, showed very high agreement with DNA methylation profiling. Its sensitivity was 100%, specificity 97%, positive predictive value 99%, negative predictive value 100%, and overall accuracy 99%. Only one false-positive classification occurred. In this case, specialist neuropathological assessment described proliferative and mitotic activity, whereas DNA methylation profiling classified the specimen as tumour-free brain tissue. Given the potential for sampling differences and intratumoral heterogeneity, this isolated discordance should be interpreted cautiously. Within this selected cohort, conventional assessment performed in a specialist neuropathology department showed high agreement with the EPIC-based reference classification for distinguishing high-grade from non-high-grade lesions.

Nevertheless, high agreement at the level of binary classification did not imply complete agreement regarding the specific tumour entity. In two cases, DNA methylation profiling refined the conventional diagnosis from a high-grade astrocytic neoplasm to glioblastoma, IDH-wildtype, CNS WHO grade 4. These cases remained true-positive high-grade gliomas in the diagnostic accuracy analysis; however, their EPIC-based tumour classifications were refined after molecular analysis. Therefore, even when specialist neuropathological assessment correctly recognises a high-grade glioma, DNA methylation profiling may provide additional diagnostic value by refining or redefining the specific glioma entity.

These diagnostic refinements should be interpreted within the evolution of CNS tumour classification. The 2016 WHO classification introduced the integration of molecular characteristics into the definition of several CNS tumour entities, an approach further expanded in the 2021 classification [19, 20]. Molecular investigations are required for the definitive classification of certain gliomas; accordingly, diagnostic changes following molecular characterisation do not necessarily indicate errors in the initial histopathological assessment.

Access to specialist neuropathological expertise and advanced molecular diagnostics remains limited in many healthcare settings worldwide, particularly in low-resource regions. Consequently, the diagnosis and classification of gliomas may still rely predominantly on histomorphological examination and immunohistochemistry, without comprehensive molecular characterisation. Although molecular parameters have become integral to contemporary WHO classification, their implementation in routine clinical practice remains heterogeneous. Our findings highlight the potential diagnostic uncertainty associated with conventional neuropathological assessment in the absence of additional molecular investigations. Such uncertainty may have clinical implications, including potentially inappropriate treatment selection, surveillance strategies, or prognostic assessment, although these outcomes were not evaluated in the present study. Where comprehensive molecular diagnostics are unavailable, referral of diagnostically challenging cases to specialist neuropathology centres, whenever feasible, may represent a practical approach to improving diagnostic accuracy.

An important distinction should be made between the clinical role of DNA methylation profiling and its use as the reference standard in the present diagnostic accuracy analysis. In routine clinical practice, DNA methylation profiling complements conventional neuropathological assessment and contributes to the final integrated diagnosis rather than replacing histopathological evaluation. In this study, EPIC-based classification alone was used as a predefined operational reference for comparing the initial conventional classifications of high-grade and non-high-grade lesions. Accordingly, false-positive and false-negative classifications indicate disagreement with this reference standard and should not necessarily be interpreted as definitive errors in the initial histopathological assessment. This distinction is particularly relevant for cases with limited tumour content, sampling heterogeneity, or histologically ambiguous findings, where additional molecular investigations would ordinarily be appropriate.

DNA methylation profiling is an important complement to conventional histopathology in diffuse gliomas and other central nervous system tumours. It can improve diagnostic precision by enabling tumour reclassification and subclassification while supporting assignment to biologically defined entities. In addition, molecularly refined classification may provide prognostic information, influence therapeutic decision-making, and contribute to greater standardisation of tumour classification by reducing interobserver variability associated with morphology-based diagnosis alone [31].

The German Cancer Research Centre (DKFZ) classifier represents an important advance in molecular neuropathology. This random forest-based classifier compares genome-wide DNA methylation profiles with a large reference cohort of central nervous system tumour entities. It provides a calibrated confidence score for each prediction [3]. Despite its growing clinical importance, DNA methylation profiling has not yet been uniformly implemented in routine brain tumour diagnostics across many healthcare systems, including centres in Europe and Germany [5, 30].

Accurate molecular characterisation has become increasingly important because modern glioma classification, prognosis, and treatment selection are closely linked to the tumour's molecular profile. For example, MGMT promoter methylation is strongly associated with response to alkylating chemotherapy and patient survival [10]. The landmark trial by Stupp et al. demonstrated that the addition of temozolomide to radiotherapy significantly improved overall survival in patients with newly diagnosed glioblastoma [28]. Subsequently, Perry et al. showed that short-course radiotherapy combined with temozolomide prolonged survival in elderly patients with glioblastoma, with treatment benefit influenced by MGMT promoter methylation status [22]. A systematic review and meta-analysis further confirmed the survival benefit associated with temozolomide-based chemoradiotherapy [9].

The therapeutic landscape of high-grade gliomas continues to evolve, encompassing optimised alkylating chemotherapy regimens and a growing range of targeted, immune-based, and cellular therapies [1, 4, 8, 11, 14, 18, 23, 25]. As treatment strategies become increasingly dependent on molecularly defined tumour subgroups, precise and timely diagnosis assumes even greater clinical importance.

Our study has several strengths. It directly compared pathological assessments performed in two diagnostic settings with different levels of neuropathological infrastructure, using EPIC-based DNA methylation classification as the operational reference standard. The independent and blinded evaluations reduced observer bias. The cohort included a range of intracranial lesions, although selection was restricted to patients with complete results from all three assessments. Consequently, the findings address a clinically relevant question regarding the diagnostic performance of conventional pathological assessment in different diagnostic settings and the additional contribution of molecular classification.

The findings have two principal clinical implications. First, intracranial tumour specimens should preferably be evaluated in specialised neuropathology centres, particularly when the initial pathological diagnosis suggests non-neoplastic tissue, nonspecific reactive changes, or a low-grade glioma despite clinical or radiological suspicion of an aggressive lesion. Second, although specialist conventional neuropathology demonstrated excellent diagnostic performance, DNA methylation profiling remains valuable because it can refine or redefine the final integrated diagnosis. DNA methylation profiling should therefore be regarded as a complementary diagnostic modality rather than a replacement for expert neuropathological assessment.

Furthermore, tissue quality, quantity, and cellular composition are important determinants of diagnostic reliability. Limited tissue availability, particularly in stereotactic biopsy specimens, or low tumour cell content may compromise both histopathological assessment and molecular classification. The presence of necrosis, haemorrhage, or substantial non-neoplastic tissue may further complicate diagnostic interpretation and contribute to discordant findings. Therefore, discrepancies between conventional neuropathology and DNA methylation profiling should not necessarily be attributed solely to differences in diagnostic techniques, as sampling variability and tissue characteristics may also play a role. These considerations emphasise the importance of obtaining representative tumour tissue and integrating morphological, immunohistochemical, and molecular findings to achieve the most reliable diagnosis.

Several limitations should be acknowledged. First, the retrospective selection of patients based on the availability of complete diagnostic results may have introduced selection bias. Although DNA methylation profiling was routinely incorporated into the diagnostic workflow at the specialist neuropathology department, inclusion required complete archived results from both conventional neuropathological assessments and DNA methylation profiling. Patients without a complete diagnostic pathway were therefore excluded. Furthermore, for some excluded cases, it could not be established retrospectively whether specimens had not been submitted, diagnostic assessments had not been performed, or the corresponding documentation was unavailable. Consequently, the reported diagnostic accuracy estimates should be interpreted within this selected cohort and may not be generalisable to all patients undergoing surgery for intracranial space-occupying lesions.

Second, the index tests and reference standard were not based on identical tissue specimens in all comparisons, potentially contributing to discordant findings through intratumoral heterogeneity or differences in tumour content between samples [17]. In particular, DNA methylation profiling was performed on the same tissue specimen used for Index Test II, whereas Index Test I was evaluated on a separate specimen. Although the Index Test II report was finalised before the DNA methylation profiling result became available, thereby providing temporal blinding, this asymmetry in specimen sharing may have favoured agreement between Index Test II and the reference standard.

Third, the study lacked longitudinal clinical follow-up and outcome data, precluding assessment of whether molecular reclassification altered treatment decisions or patient prognosis. Finally, we did not evaluate costs, turnaround times, technical requirements, or the feasibility of routine DNA methylation profiling. These considerations are important when integrating specialist neuropathology and molecular diagnostics into routine clinical workflows and should be addressed in future prospective studies.

Fourth, DNA methylation profiling was used as the operational reference standard for the diagnostic accuracy analysis but should not be considered an infallible diagnostic method. Its performance may be influenced by tumour cell content, tissue quality, and the representation of specific tumour entities within the reference classifier. Therefore, discordance between conventional neuropathology and DNA methylation profiling does not necessarily establish that the conventional diagnosis was incorrect. Ideally, such discrepancies should be resolved through integrated assessment incorporating histopathology, immunohistochemistry, molecular findings, and clinical and radiological information. Consequently, the reported diagnostic accuracy estimates represent agreement with the predefined methylation-based classification rather than absolute diagnostic correctness.

## Conclusions

In conclusion, conventional pathological assessment performed in a general pathology setting without access to comprehensive neuropathological molecular diagnostics may fail to identify a clinically relevant proportion of high-grade gliomas. In contrast, assessment in a specialist neuropathology setting showed high agreement with EPIC-based classification. Intracranial tumour specimens should therefore preferably be evaluated in specialised neuropathology centres, particularly when pathological findings are discordant with the clinical or radiological presentation. DNA methylation profiling provides additional diagnostic value by refining the molecular classification of gliomas and should be considered complementary to, rather than a replacement for, expert neuropathological assessment.

## Data Availability

No datasets were generated or analysed during the current study.
